# An Inspection Technique for Steel Pipes Wall Condition Using Ultrasonic Guided Helical Waves and a Limited Number of Transducers

**DOI:** 10.3390/ma16155410

**Published:** 2023-08-02

**Authors:** Renaldas Raišutis, Olgirdas Tumšys, Egidijus Žukauskas, Vykintas Samaitis, Lina Draudvilienė, Audrius Jankauskas

**Affiliations:** 1 Ultrasound Research Institute, Kaunas University of Technology, K. Baršausko St. 59, LT-51423 Kaunas, Lithuania; 2 Department of Electrical Power Systems, Faculty of Electrical and Electronics Engineering, Kaunas University of Technology, Studentu g. 50, LT-51368 Kaunas, Lithuania

**Keywords:** pipeline corrosion, non-destructive testing, defect detection, ultrasonic guided waves, finite element modeling, phase delay, helical wave signal

## Abstract

This research utilizes Ultrasonic Guided Waves (UGW) to inspect corrosion-type defects in steel pipe walls, providing a solution for hard-to-reach areas typically inaccessible by traditional non-destructive testing (NDT) methods. Fundamental helical UGW modes are used, allowing the detection of defects anywhere on the pipe’s circumference using a limited number of transducers and measurements on the upper side of the pipe. Finite element (FE) modeling and experiments investigated generating and receiving UGW helical waves and their propagation through varying corrosion-type defects. Defect detection is based on phase delay differences in the helical wave’s signal amplitude peaks between defective and defect-free regions. Phase delay variations were noted for the different depths and spatial dimensions of the defects. These results highlight the phase delay method’s potential for NDT pipeline inspection.

## 1. Introduction

The pipelines used in oil and gas infrastructures are subject to corrosion, leading to a gradual decrease in wall thickness and potentially causing leakage of hazardous substances. For instance, the global economic impact of corrosion was estimated at US$2.5 trillion, which was equivalent to 3.4% of global GDP in 2013 [1]. The EMARS database reports 137 major refinery accidents in EU countries since 1984, with approximately 20% of these accidents indicated as being caused by corrosion [2]. To prevent severe environmental and economic consequences, periodic inspection of corrosion rates is essential for maintaining the long-term integrity of these assets. It is estimated that corrosion prevention practices, including non-destructive testing, can reduce annual corrosion-related costs by 15–35%. This leads to significant savings in the oil, gas, and even aerospace sectors [1,3].

Ultrasonic testing methods have emerged from conventional techniques such as polarization resistance [4] and weight loss measurements [5] as a direct and non-intrusive approach for detecting both internal and external corrosion. Alongside visual inspection, ultrasonic testing is considered the method of choice for assessing metal loss [6]. Two major groups of ultrasonic inspection can be outlined: point-wise and long-range. Point-wise inspection is a well-established technique based on back-wall echo measurements, which currently can assess the corrosion rates of 0.1–0.2 mm/year within 1–2 h [7,8,9]. While these methods are well suited to cases where the locations of corrosion can be predicted, their surface coverage is essentially limited, which may lead to ineffective inspections. Additionally, these techniques entail considerable operational costs and require explicit access to the corroded areas. Although point-wise inspections are slow, their sensitivity to defects is of an order that no other technique can currently achieve. Classical long-range measurements are based on ultrasonic guided waves (UGW), which take advantage of low-frequency fundamental modes to inspect large distances from a single transducer position. Significant progress has been demonstrated in the application of UGW for corrosion detection in pipes [10], rebars [11], and steel strands [12]. Conventional long-range UGW inspection is known to suffer from limited measurement sensitivity. Therefore, there has been growing interest in the use of the constant group velocity method [13], guided wave tomography [14,15], and high order modes [16], trading the inspection area for improved detectability. The constant group velocity method estimates average wall thickness loss using a flexural mode propagating between a pair of guided wave transducers located at a distance of several pipe diameters apart. The frequency of the fundamental flexural mode is selected so that the group velocity of the mode remains constant over the range of wall thickness variation. Meanwhile, the phase velocity is highly dispersive, allowing for the estimation of the remaining wall thickness from phase angle measurements [17]. While this method can smoothly integrate wall thickness over propagation length, it fails to capture thickness variations and localized defects. 

Spatially localized inhomogeneities can be detected using UGW tomography, which is an effective way to reconstruct the spatial distribution of wave velocity, attenuation, frequency shift, or damage index from the projection data, which can be associated with the presence of defects [18,19,20,21,22]. The principle of tomography requires gathering a large number of projections. Hence, the accuracy of the reconstruction can be improved by increasing the number of propagation paths and exploiting helical waves with different numbers of turns around the pipe. Helical modes ensure better ray coverage around the circumference of the pipe and artificially expand the aperture of the array [15,23,24]. 

On the other hand, the accuracy of tomographic reconstruction relies on the number of insonification angles. In the case of cylindrical structures, this leads to arrays surrounding the circumference of the pipe. If only a portion of the tested pipe could be accessed, clamping a full ring of transducers may create an inconvenience. For this reason, UGW screening techniques have emerged. These techniques measure the travel time of directional dispersive waves traveling around the circumference of the pipe [16]. With the transducers mounted on the top surface of the pipe, accessibility requirements can be partially mitigated. The reduced inspection distance around the circumference has allowed for the implementation of high-order modes for increased inspection sensitivity, such as A1 or SH1 [25,26,27,28]. However, existing structural features of the pipelines, such as bends, joints, junctions, or flanges, inevitably cause additional scattering and generation of co-existing modes, which essentially complicates the analysis of inherently difficult high-order UGW signals. Therefore, it is crucial to develop, investigate, and propose a technique that can effectively detect concentrated defects at any location along the pipe circumference while utilizing a limited number of transducers and employing simplified signal analysis.

The aim of the presented work is to propose the application of fundamental helical UGW modes as a technique for defect detection. Additionally, the research aims to investigate the feasibility of using these waves to detect defects at any location along the pipe’s circumference by employing a limited number of transducers. Furthermore, the study aims to explore the interaction effects between these waves and corrosion-type defects of varying depths and spatial dimensions. Lastly, the research seeks to propose a helical wave parameter that can be utilized for defect characterization.

The paper is organized as follows: The object under investigation (the pipe) and inspection challenges are presented in Section 2. A description of the models and modeling results of UGW propagation along the pipe has been presented in Section 3. Results of the experimental verification of UGW generation and propagation have been presented in Section 4. The selection and analysis of helical wave signals are presented in Section 5. An investigation of helical wave interaction effects with corrosion-type defects possessing different depths and spatial dimensions has been presented in Section 6, followed by conclusions in Section 7.

## 2. The Object under Investigation

Corrosion in steel pipelines can have significant consequences, particularly in the lower part where accumulated sediments, moisture, and temperature variations can accelerate the process of wall thickness reduction, thereby damaging the pipeline’s structural integrity. Corrosion in the lower part of the pipeline typically possesses significant challenges for NDT inspection and maintenance activities. For example, accumulated sediments create significant limitations for visual inspections and the need for specialized inspection techniques can increase operational costs. The presence of dirt, rust, or accumulated sediments on the lower part of the pipeline can complicate the application of NDT methods that depend on surface conditions, such as uneven or contaminated surfaces. In some cases, the pipeline may be supported by concrete or metallic structures, complicating the assessment of the pipe’s surface in those regions. 

**Figure 1:**
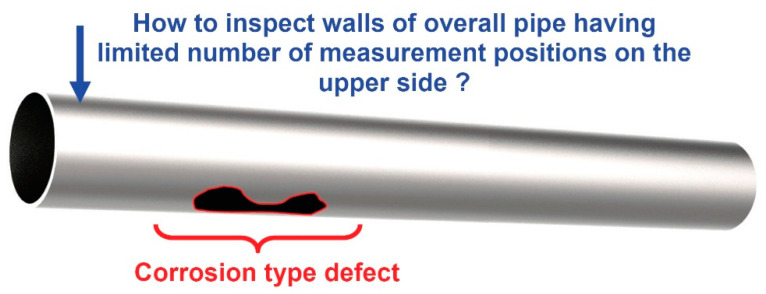
The graphical representation of the object under investigation (steel pipe) and NDT inspection challenges.

To overcome these challenges, it is essential to propose the most suitable NDT method for effective overall pipeline inspection using a limited number of measurement positions, including the lower part. The graphical representation of the object under investigation and inspection challenges is presented in Figure 1.

The UGW technique, based on the utilization of fundamental helical modes, shows promise as a tool for detecting concentrated defects at any point along the pipe circumference. This technique employs a limited number of transducers and simplified signal analysis, bridging the gap between point-wise inspection and long-range measurements. The approach involves scanning the receiving transducer in the axial direction of the pipe and utilizing helical waves to detect defects located at various circumferential and axial positions. By leveraging the sensitivity of helical waves to concentrated defects and the long propagation distances offered by fundamental modes, this technique strikes a balance between accurate point-wise measurements and long-range coverage. Notably, the proposed technique does not necessitate direct access to corroded areas, yet it provides substantial inspection coverage.

An analysis of available UGW modes and propagating effects within the steel using appropriate semi-analytical finite element and finite element methods is presented in the next section.

## 3. Modeling of UGW Propagation in a Steel Pipe

**Figure 2:**
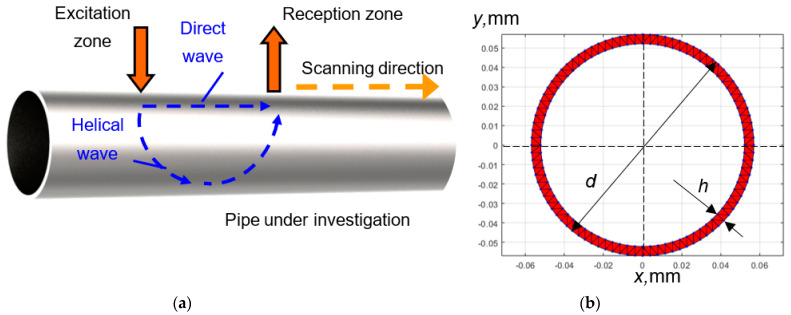
Graphical representation of the FE numerical model for the investigation of UGW propagation (direct and helical wavepaths) along the defect-free pipe (**a**) and mesh of the cross–section of the steel pipe being investigated using the SAFE method (**b**).

Numerical simulation was performed to study the propagation of UGW in a defect-free pipe. A simplified graphical representation of the model is shown in Figure 2a. The steel material properties were used for calculation: Young‘s modulus *E* = 207 GPa, Poisson‘s ratio *ν* = 0.3, and density *ρ* = 7850 kg/m^3^. The outer diameter *d* of the pipe being analyzed is *d* = 114 mm and the wall thickness *h* = 4.8 mm (Figure 2b). 

In order to perform a theoretical estimation of UGW propagation along the steel pipe, it is necessary to calculate dispersion curves. The dispersion curves of UGW propagating in a steel pipe were calculated using the Semi-Analytical Finite Element (SAFE) method [29]. In order to select the diameter of the pipe being investigated (whether smaller or larger), the smaller diameter pipe was chosen. Testing and modeling on smaller diameter pipes offer practical advantages, such as the feasibility of constructing a laboratory set-up and the experimental control of a reduced dimensional object. It also provides essential time efficiency when conducting faster finite element modeling of various wavepaths (e.g., direct, helical) and validating different models before applying them to larger diameter pipes. 

**Figure 3:**
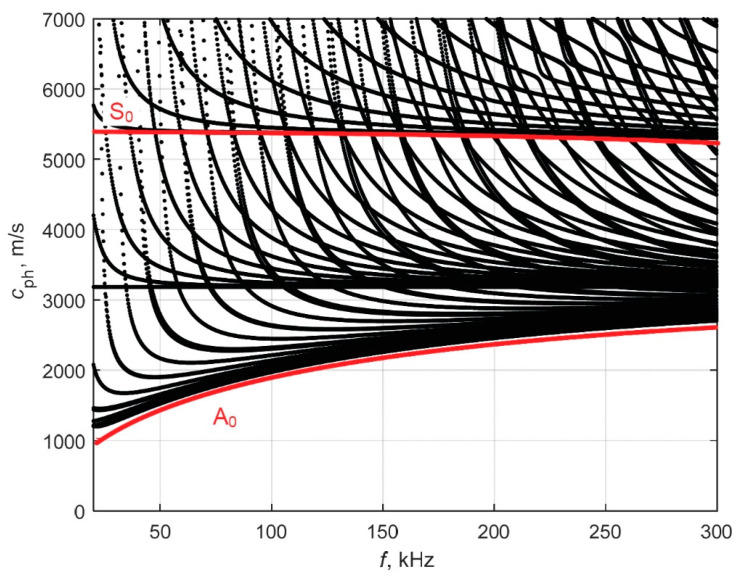
Phase velocity dispersion curves of UGW for a 4.8 mm thickness steel pipe (black lines), calculated using the SAFE method and comparison of dispersion curves calculated for the plate (red lines).

The SAFE input mesh of the pipe cross-section is shown in Figure 2b. The obtained dispersion curves of guided waves in the 20–300 kHz frequency range are presented in Figure 3 (black lines). It can be seen that within the frequency range, a high number of possible modes can propagate. However, the literature analysis revealed that when the ratio of the pipe’s wall thickness and radius is less than 20%, the dispersion curves of a hollow cylinder are similar to the dispersion curves of Lamb waves propagating in a plate [30,31]. In our case, the ratio of wall thickness to pipe radius is 8.4%. Dispersion curves of the Lamb waves of a 4.8 mm thickness steel plate were calculated and presented in Figure 3 (red lines). These curves show that in the frequency range of interest, only fundamental Lamb wave modes (A_0_ and S_0_) propagate. The asymmetric A_0_ mode was selected for investigation due to its dominant out-of-plane displacement component, which ensures more efficient excitation and reception when using contact-type ultrasonic transducers operating in thickness mode. Additionally, at lower frequencies, the A_0_ mode exhibits dispersion, and the phase velocity of this mode is reduced due to the thickness reduction caused by corrosion. The reduction in phase velocity across the defective region will provide valuable information about the presence and characteristics of the corrosion defects. The phase velocity value of the A_0_ mode at 150 kHz is 2130 m/s.

**Figure 4:**
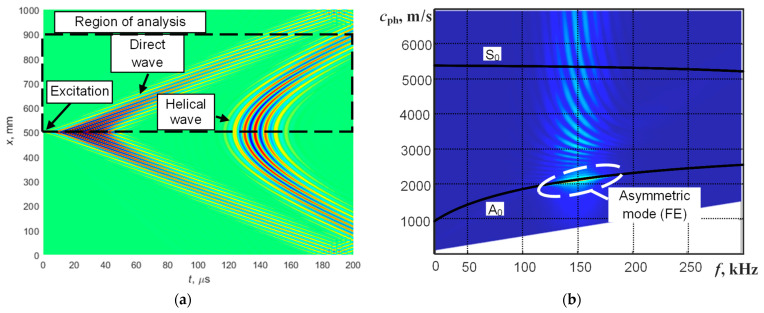
The simulated B-scan image of direct and helical UGW propagation along the defect-free steel pipe (**a**) and comparison of reconstructed dispersion curves of UGW propagation in the steel pipe (FE modeling and dispersion curves were reconstructed using the 2D FFT method) with calculated dispersion curves of a steel plate (SAFE technique) (**b**). The A_0_ and S_0_ modes are marked for the solid line curves calculated using SAFE and the reconstructed segment (using 2D FFT) of the propagating asymmetric mode is marked by a white dashed contour. The colormap represents amplitude in arbitrary units.

To investigate the propagation effects of UGW in a defect-free pipe (along and across the pipe), numerical modeling of UGW (Lamb waves) propagation in a steel pipe was performed using the “Abaqus” finite element (FE) software (Dassault Systèmes, Vélizy-Villacoublay, France). The length of the pipe being analyzed is 1000 mm. The 3D model was meshed using C3D8R brick elements. The size of the element was 1 mm and corresponds to 14 elements per wavelength of the A_0_ Lamb wave mode at 150 kHz frequency. UGW (mainly the A_0_ mode) in the pipe were excited using a normal incidence force applied at the center of the analyzed pipe segment, specifically at an axial distance of 500 mm. Such an excitation distance from both free ends of the pipe allows for the avoidance of unwanted effects of reflections of the propagating UGW (Lamb waves). The spatial size of the excitation zone was 2 mm × 2 mm. This spatial zone, which is smaller than half a wavelength, corresponds to point-type excitation and produces a circularly shaped directivity pattern. The excitation signal had a frequency of 150 kHz and a 5-period sine burst with a Gaussian envelope. Particle velocities of propagating Lamb waves were collected along the B-scan line intersecting with the excitation point (Figure 2a). Only the out-of-plane component was recorded for analysis because this component is the dominant component of the asymmetric wave mode. The simulated B-scan image of direct and helical wave propagation along the defect-free steel pipe is presented in Figure 4a. For the first attempt, a single turn of helical-guided waves around the pipe was analyzed instead of multiple turns. This was done to avoid reducing ambiguity, interference, and the overlapping of guided waves.

Dispersion characteristics of the phase velocity of propagating UGW from the simulated B-scan image can be obtained using a two-dimensional Fourier transform (2D FFT) [32]:

(1) $$H(k,f)=\int_{-\infty }^{+\infty }\int_{-\infty }^{+\infty }u(x,t)e^{-j(kx+\omega t)}dxdt$$

(2) $$c_{ph}=\frac{\omega }{k}$$

where *x* is the coordinate, *t* is the time, ω = 2π*f* is the angular frequency, *k* is the wavenumber, and *c*_ph_ is the phase velocity. The reconstructed dispersion curves using 2D FFT, in comparison with theoretical dispersion curves obtained using the SAFE technique, are presented in Figure 4b. The perfect coincidence of dispersion characteristics shows that UGW excited using point-type excitation in the pipe with a low ratio of wall thickness to radius are sufficiently similar to UGW propagating in plates with the same thickness.

In order to verify the modeling results and determine the propagating UGW within the pipe, it is necessary to perform experimental investigations. The experimental set-up and results are presented in the next section.

## 4. Experimental Verification of UGW Generation and Propagation along and around the Pipe

To verify the simulation results related to direct and helical wavepaths of UGW, the experimental investigation of UGW generation and propagation along a steel pipe with an external diameter of 114 mm and a wall thickness of 4.8 mm was performed. The length of the pipe was 3000 mm.

**Figure 5:**
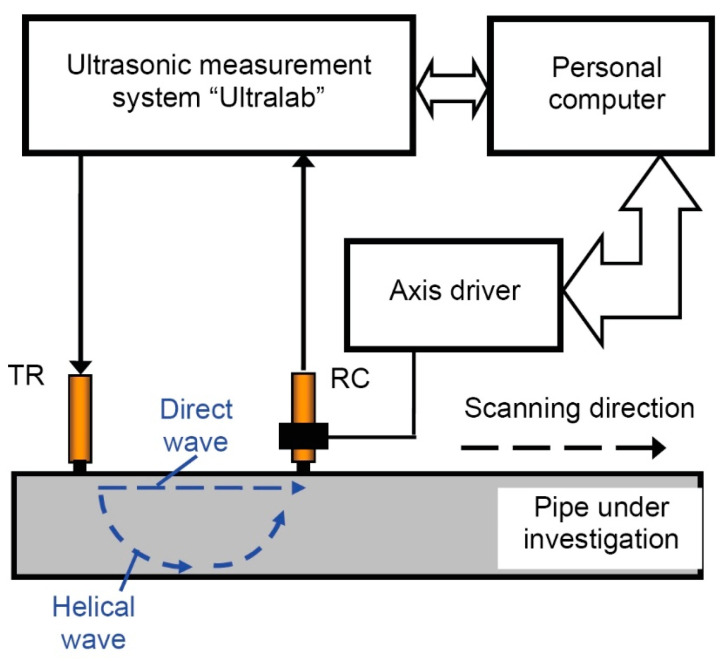
Experimental set-up for the inspection of the steel pipe using direct and helical waves.

The ultrasonic low frequency measurement and data acquisition system “Ultralab”, developed at the Ultrasound Research Institute of Kaunas University of Technology, was used for the generation and registration of propagating UGW (Lamb waves) signals (Figure 5). Contact-type transmitting and receiving low-frequency ultrasonic transducers, possessing point-type spherical working surfaces, were used for the excitation and reception of UGW. The transducers’ bandwidth ranged from 40 kHz to 640 kHz (at −10 dB) [33].

The transmitter was fixed on the surface of the pipe, in the middle of the axial length of the pipe. The initial distance between the transmitter and receiver was 100 mm. The transmitter was excited by a 3-period burst Gaussian envelope signal with an amplitude of 300 V. The receiver was scanned by a linear mechanical scanner along the pipe up to 400 mm away from the transmitter with a scanning step of 0.5 mm. The sampling frequency of the analog-to-digital converter for digitizing the received signals was 12.5 MHz.

**Figure 6:**
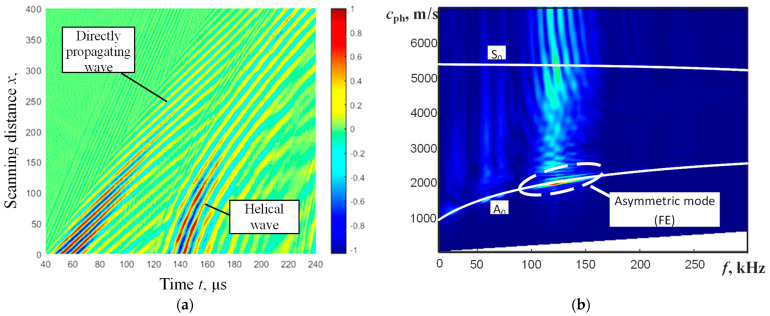
Experimentally obtained B-scan image of direct and helical wave (**a**) and reconstructed phase velocity dispersion curves (**b**) using the 2D FFT technique and calculated theoretical solid line curves of the A_0_ and S_0_ modes by the SAFE method. The colormap represents amplitude in arbitrary units.

The experimentally obtained B-scan image of propagating UGW in direct and helical wavepaths and reconstructed phase velocity dispersion curves using the 2D FFT technique are presented in Figure 6a,b. To compare the obtained result with the theoretical phase velocity dispersion curves of the A_0_ and S_0_ modes for the steel plate, the calculated curves using the SAFE method are also presented (Figure 6b). 

The phase velocity value of the A_0_ mode from the experimentally measured B-scan image was found to be 2115 m/s at 150 kHz. From calculations using the SAFE method, the phase velocity of the A_0_ mode is 2130 m/s. This suggests a good coincidence between the simulated and measured results.

In the subsequent section, the selection and analysis of the helical wave signals are presented, as the focus of this work lies in utilizing helical UGW propagating around the circumference of the pipe for defect detection and parameterization.

## 5. Selection and Analysis of Helical Wave Signals

**Figure 7:**
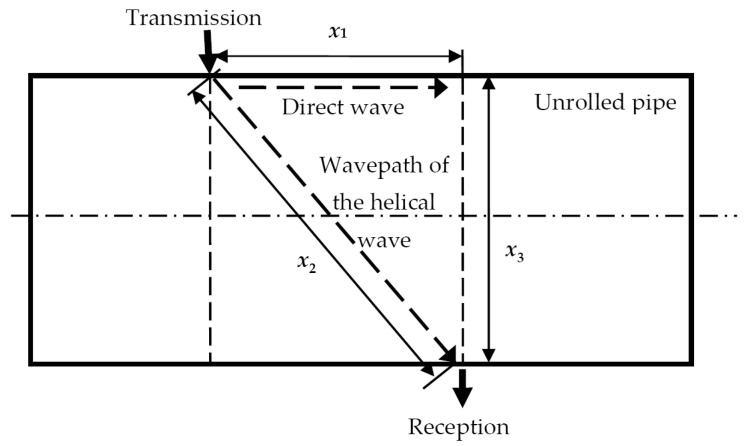
Wavepath of the direct wave (*x*_1_) and wavepath around the pipe (helical wave, *x*_2_) within the unrolled pipe, where *x*_3_ is the arc length of the pipe circumference and *d* is the diameter of the pipe.

To effectively use helical UGW propagating around the circumference of the pipe for defect detection and parameterization, it is necessary to correct the non-linear helical distance of the UGW by recalculating it to the linear distance along the scanning direction. The wavepath of the direct wave (*x*_1_) and the wavepath around the pipe (helical wave, *x*_2_) within the unrolled pipe are presented in Figure 7.

The different propagation trajectories of UGW (wavepaths *x*_1_ and *x*_2_) are interrelated:

(3) $${x_{2}}^{2}={x_{1}}^{2}+{x_{3}}^{2}$$

where *x*_1_ is the direct wavepath distance of UGW between the fixed transmitter and moving receiver, *x*_2_ is the helical wavepath distance along the circumference of the pipe, *x*_3_ = π*d* is the arc length of the pipe circumference, and *d* is the pipe diameter.

For the selection and analysis of helical wave signals, the B-scan image simulated by FE (described in Section 3 and presented in Figure 4a) was selected. UGW propagation trajectories for the direct wave and helical wave within the B-scan image are determined from the maxima of the signal envelopes:

(4) $$x_{1,k}\left(t_{1,k}\right)=max\left(HT\left[u_{1,k}\left(t\right)\right]\right)$$

(5) $$x_{2,k}\left(t_{2,k}\right)=max\left(HT\left[u_{2,k}\left(t\right)\right]\right)$$

where *u*_1,*k*_(*l_k_*, *t*) and *u*_2_,*_k_*(*l_k_*, *t*) are signals of the direct and helical waves with propagation distances *x*_1_ and *x*_2_, *k* is the current scanning point number along the pipe (*k* = 1…*N*, *N* is the number of scanning points along the pipe), and HT is the Hilbert transform for the calculation of the signal envelope.

**Figure 8:**
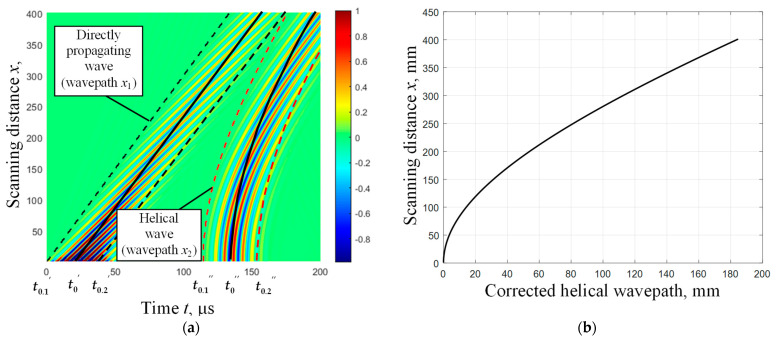
Simulated B-scan image of direct and helical waves (**a**) propagation along the steel pipe. The determined peak values of the direct and helical UGW signal envelopes are presented by solid lines at the initial scanning point (${t_{0}}^{\prime }$ and ${t_{0}}^{\prime \prime }$). The boundaries of time-domain windows for the selection and analysis of signals are presented by dashed lines (for the direct wave ${t_{0.1}}^{\prime }$, ${t_{0.2}}^{\prime }$ and for the helical wave ${t_{0.1}}^{\prime \prime }$, ${t_{0.2}}^{\prime \prime }$). The dependence between the actual scanning distance of the receiving transducer on a linear scale and the distance after correcting the helical wavepath (**b**) by recalculating it to the distance along the scanning direction of the pipe (using Equation (10)).

As seen in Figure 8a, in the case of direct UGW propagation (wavepath *x*_1_), the interdependence between the scanning distance *x* and propagation time *t* is linear. However, in the case of helical UGW propagation (wavepath *x*_2_), this interdependence is nonlinear. 

Therefore, in order to perform detection and reliable non-destructive evaluation of defects located on the lower part of the pipe, it is necessary to develop an algorithm to correct the nonlinear dependence to a linear one. The dependency presented in Equation (3) was used for the development of this algorithm.

If we assume that the group velocity *c*_gr_ of propagating UGW is the same along the wavepaths of the direct wave *x*_1_ and the helical wave *x*_2_, then it can be written:

(6) $$c_{gr}=\frac{x_{1}}{t^{\prime }}=\frac{x_{2}}{t^{\prime \prime }}.$$

where $t^{\prime }$ and $t^{\prime \prime }$ are the propagation times of the UGW signals’ envelope peaks, along the direct wavepath and helical wavepath, respectively.

The group velocity can be calculated from the direct propagation of UGW signals between the transmitting and receiving transducers (along the direct wavepath *x*_1_):

(7) $$c_{gr}=\frac{{x_{1,N}-x}_{1,1}}{t_{N}^{\prime }-t_{0}^{\prime }},$$

where *x*_1,1_ is the initial scanning position of the receiver and $t_{0}^{\prime }$ is the corresponding time point of the direct wave signal envelope peak, *x*_1,N_ is the farthest scanning point of the receiver and the corresponding time point $t_{N}^{\prime }$ of the signal envelope, and *N* is the total number of receiver scanning points.

Combining Equations (3) and (6) yields:

(8) $${c_{gr}}^{2}\cdot t_{r}^{\prime \prime 2}={c_{gr}}^{2}\cdot t^{\prime 2}+{\left(x_{3}\right)}^{2},$$

(9) $$t_{r}^{\prime \prime }=\sqrt{t^{\prime 2}+{\left(x_{3}/c_{gr}\right)}^{2}}.$$

where $t_{r}^{\prime \prime }$ is the propagation time of the helical wave along wavepath *x*_2_ and $t^{\prime }$ is the propagation time of the direct wave along wavepath *x*_1_.

The correction of the non-linear helical distance of the propagating UGW is achieved by recalculating it to the linear distance along the scanning direction and compensating for the arc length *x*_3_ of the pipe circumference:

(10) $$x_{2r}\left(t_{r}^{\prime \prime }\right)=c_{gr}\cdot t_{r}^{\prime \prime }-x_{3},$$

According to Equation (10), it is possible to estimate the distance along the scanning direction of the pipe that corresponds to the UGW propagation distance in the helical trajectory. The derived dependence is illustrated in Figure 8b.

**Figure 9:**
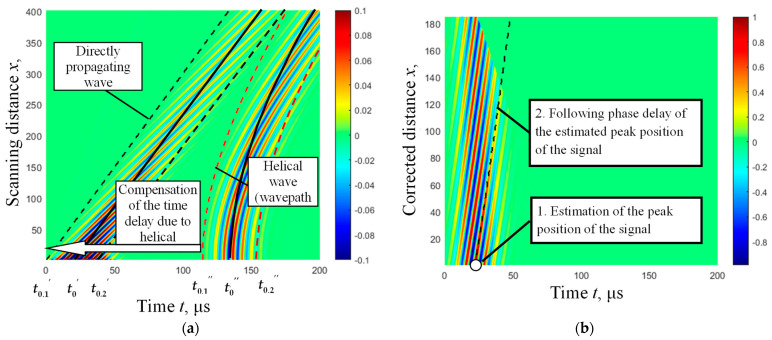
The simulated B-scan images of propagating direct and helical waves along the defect-free pipe: (**a**)—the B-scan image of the direct and helical wavepaths. The determined peak values (${t_{0}}^{\prime }$ and ${t_{0}}^{\prime \prime }$) of the direct and helical UGW signal envelopes are presented by solid lines at the initial scanning point. The boundaries of time-domain windows for the selection and analysis of signals are presented by dashed lines (${t_{0.1}}^{\prime }$, ${t_{0.2}}^{\prime }$ and ${t_{0.1}}^{\prime \prime }$, ${t_{0.2}}^{\prime \prime }$), (**b**)—the B-scan image of the selected helical wave and the recalculated helical wavepath distance to the distance along the scanning axis. The phase delay values of the signal amplitude peak of the helical wave are presented by the dashed line.

Further analysis includes the selection of the helical wave signal using a Hanning window, defined by $t_{k,1}^{\prime \prime }$ and $t_{k,2}^{\prime \prime }$, and the compensation of the delay time due to UGW propagation along the helical wavepath (Figure 9a):

(11) $${u_{2c,k}\left(t-(t_{k,1}^{\prime \prime }:t_{k,2}^{\prime \prime })\right)=u}_{2,k}(t_{k,1}^{\prime \prime }:t_{k,2}^{\prime \prime })$$

The processed B-scan image is constructed using helical wave signals *u*_2*c*,*k*_, which have a compensated delay time (according to Equation (11)) and a recalculated helical wavepath distance (according to Equation (10)) to the linear distance along the scanning direction (Figure 9b).

To observe and detect the effect of UGW phase delay at a particular point of the *k*-th signal *u*_2*c*,*k*_ along the scanning axis due to the presence of pipe wall defects, it is proposed to determine the positive peak amplitude of the signal *u*_2*c*,*k*_ at the initial scanning position (*k* = 1) between the transmitter and receiver. Furthermore, for other signals acquired along the scanning axis at each scanning point, the determination of the phase delay of such a particular amplitude peak has been performed (Figure 9b).

From the presented results, it is evident that such phase delay consistently increases without variations along the scanning direction of the defect-free pipe.

Investigations of helical wave interaction effects with corrosion-type defects possessing different depths and spatial dimensions also observed variations of phase delays of such waves propagated through defective regions and are presented in the next section.

## 6. Modeling the Interaction Effects of Helical Waves with Corrosion-Type Defects of Varying Depths and Spatial Dimensions

**Figure 10:**
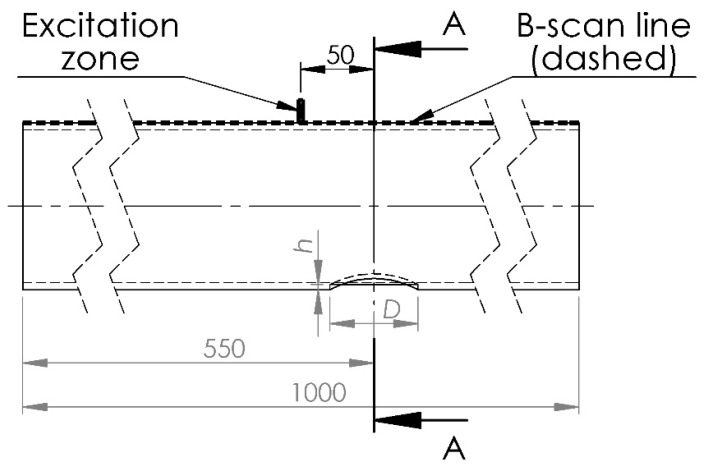
Graphical representation of the FE numerical model and longitudinal cross-section of the pipe used for investigating UGW propagation through different spatially localized defects in the pipe, where *D* is the diameter of the simulated corrosion defect and *h* is the depth of the defect. All dimensions are provided in mm. “A” represents the cross-section perpendicular to the center of the defect.

Within this section, an investigation using FE modeling is conducted to examine the interaction effects of helical waves with spatially localized corrosion-type defects (Figure 1) of varying depths and spatial dimensions. The graphical representation of the FE numerical model used for investigating UGW propagation through different spatially localized defects on the lower part of the pipe is presented in Figure 10. The defect is located on the opposite side of the pipe according to the positions of the excitation zone and receiving points of the B-scan line.

Due to the effect of phase velocity reduction of the asymmetric A_0_ mode over the corrosion-type defect with a thinner wall thickness, it is expected that due to lower phase velocity, the occurred phase delay of the UGW helical wave signal will be quantitatively measurable and allow for the detection of defects with varying depths and spatial dimensions. 

Using the SAFE method (as presented previously in Section 3), calculations of the theoretical phase velocity values within the dispersion curve of the asymmetric A_0_ mode (at 150 kHz frequency) was performed for different thicknesses of the pipe wall affected by corrosion defects (Table 1). 

This illustrative example demonstrates that a reduction in the thickness of the pipe wall will result in a lower phase velocity of propagating asymmetric UGW (in this case, the A_0_ mode) and a longer propagation time, leading to a delay in the received signal. This assumption will be verified by FE modeling and analyzing the interaction of helical UGW with different corrosion defects of varying spatial dimensions and depths.

The model parameters used are the same as those described for the previously discussed defect-free pipe in Section 3.

To investigate the possibility of detecting different depths of defects with the same diameter of 30 mm, as well as defects with different diameters (40 mm and 60 mm) and the same depths, four different defect configurations were simulated using FE modeling (Table 2). The phase delays of the UGW signal peaks were analyzed for each case. 

**Figure 11:**
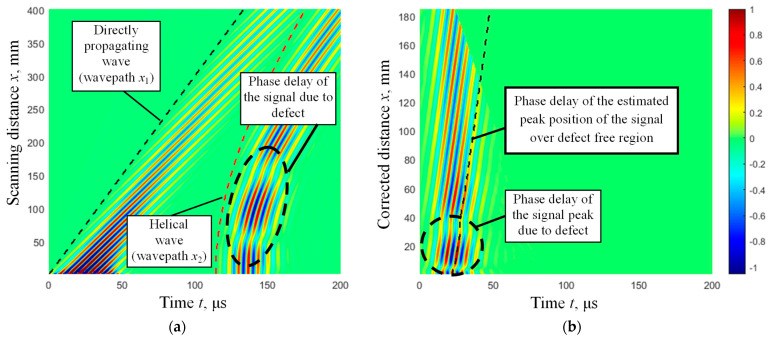

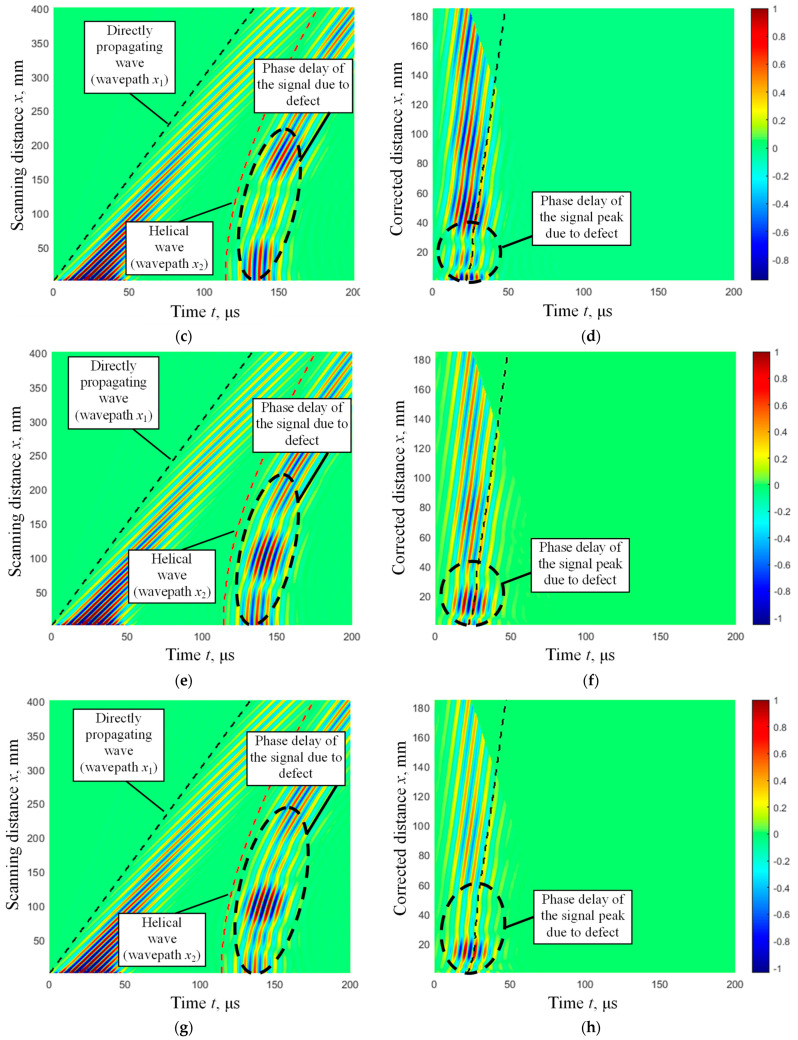
The simulated B-scan images of propagating direct and helical waves through the circularly shaped defects of different diameters and depths (Table 2). The B-scan images show the direct and helical wavepaths (**a**,**c**,**e**,**g**), also selected helical waves and the corrected helical wavepaths (**b**,**d**,**f**,**h**): (**a**,**b**)—defect 30 mm (depth 25%), (**c**,**d**)—30 mm (75%), (**e**,**f**)—40 mm (25%) and (**g**,**h**)—60 mm (25%). The phase delay values of the signal amplitude peak of the helical wave are presented by a dashed line.

**Figure 12:**
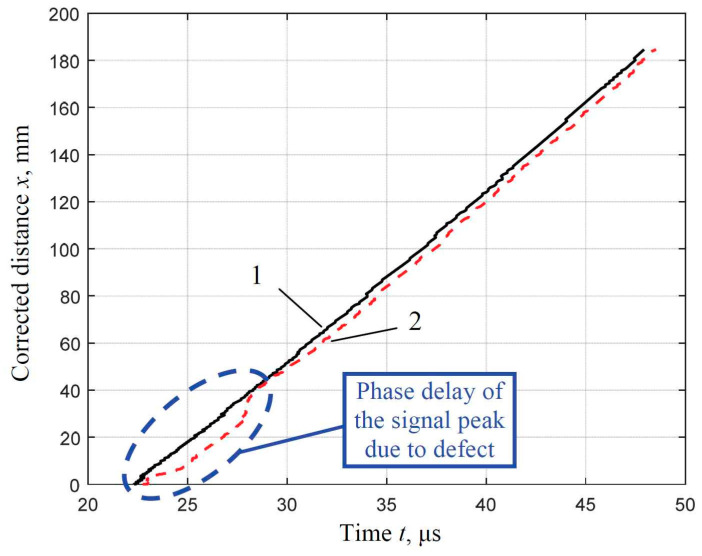
Phase delay values of the amplitude peaks of the propagated helical wave signals versus the corrected helical wavepath along the scanning distance: 1—the defect-free case (solid line); 2—the defective (diameter of the circularly shaped defect 30 mm, depth of the defect is 25% of the wall thickness).

From the results presented in Figure 11b,d,f,h, it is clearly visible that the phase delay values of the signal amplitude peaks of the helical wave are higher over the defective regions. To illustrate this measurable effect, the phase delay values of the peaks of the propagated helical wave signals (for the defect-free pipe and the defective pipe with a 30-mm defect) versus the recalculated helical wavepath to the distance along the scanning direction of the pipe are presented in Figure 12.

According to the obtained results, it is proposed to exploit this difference in phase delay values for the detection of spatially localized defective regions. For the detection and parametrization of defects with different depths and spatial dimensions, it is proposed to calculate the differences between the phase delays of the signal peaks estimated at the initial point of the receiver scanning for defect-free and defective pipes and followed along the scanning direction of the pipe.

**Figure 13:**
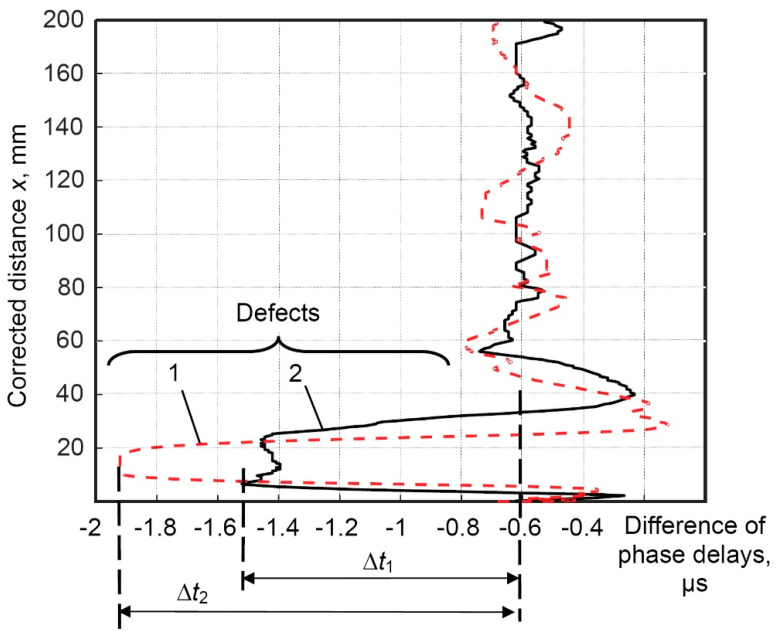
Dependence of the difference in phase delays of the propagated helical wave through the defective region by a circularly shaped defect with a diameter of 30 mm and different depths (75% and 25% of wall thickness) compared to the defect-free pipe: 1—depth of 75% of wall thickness (red dashed line), 2—depth of 25% of wall thickness (black solid line).

The dependence of the difference in phase delays of the propagated helical wave through the defective region with a circularly shaped defect with a diameter of 30 mm and different depths (75% and 25% of wall thickness) compared to the defect-free pipe is presented in Figure 13.

The calculated mean value of the initial difference in phase delays of helical waves transmitted along the completely defect–free pipe and the pipe affected by corrosion defects of different depths with a diameter of 30 mm (75% and 25% of wall thickness) in the defect-free region is −0.6 µs (Figure 13).

The differences in phase delays of helical waves at regions affected by defects, after compensation of the initial difference in phase delays (−0.6 µs), are as follows:

Δ*t*_1_ = −1.5 µs − (−0.6 µs) = −0.9 µs (depth of the defect is 25% of wall thickness);

Δ*t*_2_ = −1.93 µs − (−0.6 µs) = −1.33 µs (depth of the defect is 75% of wall thickness).

The obtained differences in phase delays of helical waves indicate the possibility of detecting defects of different depths and quantitatively differentiating their depths.

**Figure 14:**
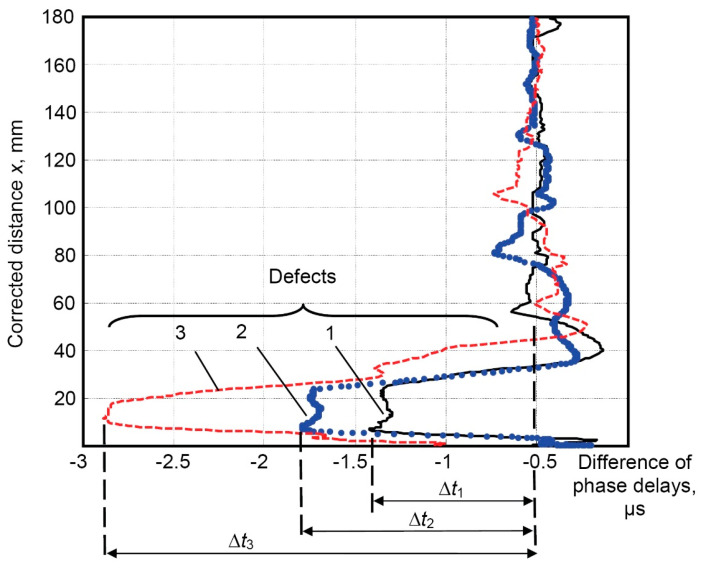
Dependence of difference in phase delays of the propagated helical wave through the defective regions of different spatial dimensions (diameters of 30 mm, 40 mm, and 60 mm), depth of all defects was 25% of wall thickness: 1—30 mm defect (black solid line), 2—40 mm defect (blue dotted line), 3—60 mm defect (red dashed line).

Dependences of the difference in phase delays of the propagated helical wave through the defective regions for circularly shaped defects with different spatial dimensions (diameters of 30 mm, 40 mm, and 60 mm) with a fixed depth of 25% of wall thickness are presented in Figure 14.

The calculated mean value of the initial difference in phase delays of helical waves transmitted along the completely defect–free pipe and the pipe affected by corrosion defects of different spatial dimensions (diameters of 30 mm, 40 mm, and 60 mm, with a depth of 25% of the wall thickness) at the defect-free region is −0.5 µs (Figure 14).

After compensating for the initial difference in phase delays (−0.5 µs), the differences in phase delays of the helical waves at the regions affected by defects are as follows:

Δ*t*_1_ = −1.4 µs − (−0.5 µs) = −0.9 µs (30 mm defect);

Δ*t*_2_ = −1.8 µs − (−0.5 µs) = −1.3 µs (40 mm defect);

Δ*t*_3_ = −2.85 µs − (−0.5 µs) = −2.35 µs (60 mm defect).

The obtained differences in phase delays of helical waves indicate the possibility of detecting defects with different spatial dimensions and quantitatively differentiating their spatial dimensions.

## 7. Conclusions

An effective technique based on the fundamental UGW helical waves is proposed for inspecting the condition of steel pipe walls. Since these helical waves are sensitive to localized defects and the UGW fundamental modes enable long propagation distances and require simple signal analysis methods, they can be used to inspect pipe wall conditions using a limited number of transducers. Furthermore, defects can be detected anywhere around the circumference of the pipe by measuring only on the pipe’s upper side. Consequently, the proposed technique does not necessitate direct access to corroded areas, yet it still provides extensive inspection coverage. The process of generating and receiving UGW helical waves was explored through both finite element (FE) modeling and experimental investigations. To demonstrate the capacity of helical UGW to detect and characterize defects, their propagation through the spatially localized defects of varying dimensions and depths was analyzed using FE modeling.

The observed difference in phase delays of the helical wave signal amplitude peaks between the defect-free region and the defective region was used for the detection and characterization of defects with varying depths and spatial dimensions. The phase delay differences obtained for a 30 mm defect with depths of 25% and 75% of the wall thickness, relative to the defect-free region, were −0.9 µs and −1.33 µs, respectively. Similarly, the phase delay differences obtained for defects with different spatial dimensions (30 mm, 40 mm, and 60 mm in diameter) and a depth of 25% of the wall thickness, relative to the defect-free region, were −0.9 µs, −1.3 µs, and −2.35 µs, respectively. The study showed a similarity in phase delay values of −1.3 µs obtained for defects with different diameters (30 mm and 40 mm) but with different depths (75% and 25% of the wall thickness, respectively). This effect can be attributed to the reduction in phase velocity of helical UGWs over the deeper defect. 

The proposed method for the quantitative evaluation of the phase delay of propagating helical UGW can be effectively used for the reliable detection and characterization of spatially localized corrosion-type defects. This technique has the potential for practical application during NDT of pipeline inspections. The results obtained suggest that a significant advantage of this method, which is based on the fundamental UGW helical waves, is that defects might be detected using multiple turns of helical waves around the pipe. This approach did not require mechanical scanning by the receiving transducer, thereby increasing efficiency.

Future research will include additional studies to better understand the observed phenomenon and the practical advantages and limitations of the developed technique. These studies will include experimental investigations on real pipe samples with different artificial defects and the real loss of wall thickness defects affected by corrosion.

## Figures and Tables

**Table 1:** Results of A_0_ mode phase velocity calculations using the SAFE method at a fixed frequency of 150 kHz for corrosion defects with different thicknesses.

Wall Thickness, mm	Depth of the Defect, mm (% of the Wall Thickness)	Remaining Wall Thickness, mm	Phase Velocity, m/s
4.8	defect-free	4.8	2130
4.8	1.2 (25%)	3.6	1936
4.8	2.4 (50%)	2.4	1666
4.8	3.6 (75%)	1.2	1247

**Table 2:** Different defect configurations used in FE simulations and obtained B-scan images.

Defect Diameter, mm	Depth of the Defect, mm (% of the Wall Thickness)	Remaining Wall Thickness, mm	B-Scan Image (Direct and Helical Waves)
30	1.2 (25%)	3.6	Figure 11a
30	3.6 (75%)	1.2	Figure 11c
40	1.2 (25%)	3.8	Figure 11e
60	1.2 (25%)	3.8	Figure 11g

## Data Availability

Due to pending patent applications, the data generated from this study is currently being kept private.
